# Causes, enablers and perceived solutions to teenage pregnancy: a qualitative study in a South-Western State in Nigeria

**DOI:** 10.11604/pamj.2022.43.120.36142

**Published:** 2022-11-03

**Authors:** Deborah Tolulope Esan, Fatimah Muhammad, Sophia Ebubechukwu Okocha, Agatha Ogunkorode, Theresa Olaitan Bamigboye, Richard Sunday Adeola, Oluwadamilare Akingbade

**Affiliations:** 1 Department of Nursing Science, College of Medicine and Health Sciences, Afe Babalola University, Ado-Ekiti, Ekiti State, Nigeria,; 2 The Nethersole School of Nursing, The Chinese University of Hong Kong, Hong Kong Special Administrative Region (SAR), Hong Kong, China,; 3 Institute of Nursing Research, Osogbo, Osun State, Nigeria

**Keywords:** Teenage pregnancy, perceived solutions, cause and enablers, South-Western Nigeria

## Abstract

**Introduction:**

teenage pregnancy remains a major public health issue in Nigeria with many teenagers being fated to early motherhood resulting in a life filled with turmoil. The aim of this study was to explore the perspectives of teachers and students of high schools on the causes, enablers and solutions to teenage pregnancy.

**Methods:**

this study employed an exploratory design using a qualitative approach. Participants were selected using purposive sampling technique and a total number of 33 participants interviewed. Data collection was done by means of audio-recorded semi-structured interviews and data were analysed using a thematic analysis approach. Descriptive statistics were used to generate participants' demographic profile.

**Results:**

three themes emerged from the study. They include perception of teachers and students on the causes of teenage pregnancy, perceived enablers of teenage pregnancy and solutions to teenage pregnancy. Findings revealed that the majority of the participants had knowledge of teenage pregnancy but had limited knowledge of contraceptives, particularly the students. Almost all participants viewed teenage pregnancy as a more common occurrence in their community than in schools. Participants identified poverty, peer pressure and poor parental control as causes of teenage pregnancy. Participants further identified poverty, substance abuse and lack of education as enablers of teenage pregnancy while perceived solutions included poverty eradication, abstinence from sexual activities, girl child education and government involvement. Majority of the participants disagreed with giving condoms to teenagers in schools.

**Conclusion:**

among other causes of teenage pregnancy identified in this study, poverty was a recurring theme. Therefore, there is, a need for the government of Nigeria to combat poverty at all levels, as a strategy to curb teenage pregnancy while not neglecting other causes.

## Introduction

In Nigeria, over 23% of teenagers aged 15-19 years have begun childbearing [1]. Early motherhood results in a role change that affects the lives of the teenage mother and child as well as family and the community they belong to. Teenage pregnancy is defined as a pregnancy that results from unprotected sexual intercourse by a girl between the age of 13-19 years [2]. Teenage pregnancy, also called unwanted pregnancy is a major public health issue globally. Despite its common occurrence, it is rarely discussed in African countries like Nigeria due to religious and cultural beliefs [3]. Unwanted pregnancy has been linked to sexual abuse, ignorance, alcohol consumption, substance abuse, cultural traditions, peer pressure and regular sexual intercourse without a reliable contraceptive [4-6]. There has been an increase in the number of deaths from criminal abortions as young girls go through treacherous measures to abort pregnancies; many researchers have reported cases of teenage girls experimenting with various dangerous concoctions in the hope of preventing or terminating unwanted pregnancy [7,8]. Furthermore, some teenage pregnancies also end in miscarriages, preterm birth, vaginal fistulas, and sometimes death [9,10]. Often times, the girl child suffers most of the consequences in the situation of unwanted pregnancy, and they are often faced with stigma from society, her peers, and family members [11]. Chiazor *et al*. [6] explained that teenage pregnancy has often been found to be a determinant of poverty as the majority of teenage mothers are without a source of livelihood to fend for themselves and their child. In Nigeria, majority of teenage mothers have to drop out of school and are unable to complete their education, which further results in lack of qualifications and future unemployment [3,4]. Coupled with an unstable financial capability, teenage pregnancy can lead to psychological and mental issues such as depression, anxiety, and emotional instability [12]. The genital stage in Freud's psychosexual theory suggests that adolescence is a stage marked by sexual exploration and experimentation; unfortunately, this experiment coupled with other factors, often leads to unplanned pregnancies [13]. A study [10] explained that some teenagers opined that early pregnancy was influenced by a desire to have a secure financial support from older men, access to child support grants and poor sexual negotiation skills. Also, in some cases teenagers reported that lack of adequate sexual knowledge was a factor that led them to early pregnancy, while some others used pregnancy as a deliberate excuse to escape the demands of high school education or to escape problems related to family [14]. This study, therefore, aims to explore the perception of students and teachers of high school on the causes, enablers and perceived solutions to teenage pregnancy in Ado-Ekiti, Ekiti State, Nigeria.

## Methods

**Study design:** this was an exploratory qualitative study using in-depth interviews, to explore the perception of teachers and students on the causes, enablers and solutions to teenage pregnancy.

**Study setting and participants:** the study was conducted between February and March 2020. It was conducted in two secondary educational institutions in Ado-Ekiti, Ekiti State. Ekiti is a State located in the Southwest region of Nigeria. This study was conducted in Christ´s high school (CHS) Ado-Ekiti and Ado Grammar school (AGS) Ado-Ekiti, both in the State Capital of Ekiti State, Southwest, Nigeria. The high schools are attended by adolescents between 9-20 years. The target population included high school students who were between 13-19 years and teachers of high schoolers between 30-60 years. A purposive sampling technique was used to select 33 participants who were made up of both teachers and students. The students and teachers range from different levels in the selected secondary schools. The inclusion criteria for this study include being adolescent students and teachers in secondary schools in Ekiti State within the age range of 13-19 years for students and 18 years and above for teachers, as well as willingness to participate in the study. A semi-structured in-depth interview was used with open-ended questions which explored the teachers´ and students´ perceptions of the causes, enablers, and perceived solutions to teenage pregnancy until data saturation was reached. Each sample group was saturated independently. A total of 33 interviews were conducted. The interviews were audiotape with the participants´ permission, transcription was done within 24 hours. Referential adequacy was attained, partially fulfilling the requirement of trustworthiness.

**Variables:** variables included teenagers, teenage pregnancy, teachers, causes, enablers, and perceived solutions.

**Sampling and sample size:** the purposive sampling method was used to select eligible participants for the study. The sample size was determined by data saturation. Thirty-three interviews were conducted.

**Data collection tool:** a semi-structured interview guide was developed as an instrument for data collection. The validity of the instrument was ensured by giving it to experts in public health and research. Also, to ensure credibility, only participants who met the inclusion criteria were interviewed. The interview was guided by questions such as knowledge of teenage pregnancy, causes of teenage pregnancy, knowledge of contraceptives, and solutions to teenage pregnancy.

**Data collection:** data collection and analysis were carried out concurrently. The students were interviewed at school during their long-break period, while the teachers were interviewed during their free time. The goals and objectives of the research were explained to all participants. The interview took an average of 30 minutes per participant. A total of 33 interviews were conducted. Data saturation was reached after conducting 30 interviews. Three more interviews were conducted without new categories evolving.

**Data analysis:** the demographic data of the participants were analyzed using the Statistical Package for Social Sciences (SPSS) version 25. Data were cleaned and checked for errors before analysis. Frequencies and percentages were calculated for demographic characteristics. Qualitative data were analyzed using a thematic analysis approach. The narrative data gathered in terms of each theme were analyzed using open coding. Data were analyzed using thematic analysis. To ensure trustworthiness, strategies such as interpersonal relationship and trust building, triangulation of data gathering methods, peer examination, member checking, dense description and dependability audit were employed.

**Ethical consideration:** permission to conduct the study was obtained from the management (principals) of the two secondary schools, after institutional approval to conduct the study was obtained from the Ethics and Research Committee of Afe Babalola University, Ado-Ekiti. Prior to the interview, each participant´s rights were explained, and written informed consent was obtained. Permission was also obtained to use an audio recorder. Informed consent was obtained from a parent and/or legal guardian for participants under 18 years. All methods were performed in accordance with the relevant guidelines and regulations.

## Results

**Demographic data:** thirty-three respondents (25 students and eight teachers) from two secondary schools in Ekiti State were involved in this study. Five teachers and 13 students were from Ado Grammar School, while three teachers and 12 students were from Christ´s high school. The response rate was 100%. All 33 interviews were subjected to analysis. Table 1 revealed the sociodemographic characteristics of the high school students that were interviewed. The participants' ages ranged from 12-18 years, with the mean age being 16 years. All participants were Christians with the majority (88%) being Yoruba. Majority (84%) of the participants were females while a few (16%) were males. All participants were single (100%). Forty-eight percent of the participants were in junior secondary class while 52% were in senior secondary class. The majority (68%) of the participants were from a monogamous family, while a few (32%) are from a polygamous family. Table 2 presents the sociodemographic characteristics of high school teachers that were interviewed. The teachers' ages ranged from 30-60 years with a mean age of 45 years. All participants were Christians and Yorubas. Majority (87.5%) were female while there was only 1 male. All participants were married (100%). Most (75%) of the participants were from a monogamous family, while a few (25%) were from a polygamous family. The summary of sociodemographic data for both students and teachers is presented in Table 3.

**Table 1:** socio-demographic data of high school students

	1	2	TOTAL
**Item**	**Ado grammar school**	**Christ's high school**	
N	13	12	25
**Average age (in years)**	13. 9	17.1	15.5=16 years
**Religion**			
Christianity	(100%)	12 (100%)	25 (100%)
**Gender**			
Male	0 (0%)	4 (33.3%)	4 (16%)
Female	13 (100%)	8 (66.7%)	21 (84%)
**Ethnicity**			
Igbo	0 (0%)	1 (18.3%)	1 (4%)
Yoruba	11 (84.6%)	11 (91.7%)	22 (88%)
Others	2 (15.4%)	0 (0%)	2 (8%)
**Marital status**			
Single	13 (100%)	12 (100%)	25 (100%)
Family type			
Monogamy	11(84.6%)	6(50%)	17(68%)
Polygamy	2(15.4%	6(50%)	8(32%)
**Class**			
JS 2-3	7(53.9%)	5(41.7%)	12(48%)
SS 1-2	6(46.2%)	7(53.9%)	13(52%)

*** JS: Junior Secondary; SS: senior secondary

**Table 2:** socio-demographic data of teachers of high school

	1	2	
**Item**	**Ado grammar school**	**Christ's high school**	**Item**
**N**	5	3	8
**Average age (in years)**	43.2	47	45.1=45 years
**Religion**			
**Christianity**	5 (100%)	3 (100%)	8 (100%)
**Gender**			
Male	1 (20%)	0 (0%)	1 (12.5%)
Female	4 (80%)	3 (100%)	7 (87.5%)
**Ethnicity**			
Yoruba	5 (100%)	3 (100%)	8 (100%)
**Marital status**			
Married	5 (100%)	3 (100%)	8 (100%)
**Family type**			6 (75%)
Monogamy	3 (60%)	3 (100%)	
Polygamy	2 (40%)	0 (0%)	2 (25%)

**Table 3:** summary of socio-demographic data of participants (both students and teachers)

Variable	N=33	%
**Socio-cultural group**		
Yoruba	30	90.9
Igbo	1	3.0
Others	2	6.1
**Religion**		
Christianity	33	100
**Gender**		
Female	28	84.8
Male	5	15.2
**Age range** 10-20	25	75.6
21-30	Nil	Nil
31-40	2	6.1
41-50	5	15.2
51-60	1	3.0

**Qualitative data:** the qualitative findings of the study are presented according to the themes and various categories generated from the data (Table 4). Each theme is described with a summary of the categories it represents. This served as a template according to which accounts from participants were calculated after the initial data analysis and the point of data saturation.

**Table 4:** main themes and subthemes from the data

Main themes	Categories
Perceptions of teachers and students on the causes of teenage pregnancy	Knowledge of teachers and students about teenage pregnancy
	Frequency of teenage pregnancy in Nigeria
	Explore the causes of teenage pregnancy
	Experience if whether pregnant teenagers get enough support from friends and families
Perceptions of teachers' and students on the enablers	Contributors to teenage pregnancy
	Knowledge of teachers and students about contraceptives
	Explore cultural practices concerning sexual experimentation
	Explore whether culture and family background encourage teenage pregnancy
Perceptions of teachers' and students on the solutions to teenage pregnancy	Opinion on what the prevention of teenage pregnancy is
	Opinion on if sharing contraceptives to teenagers is a good idea
	Possible solutions to prevent teenage
	pregnancy in Nigeria
	Explore if it advisable to talk on sex education to teenagers

**Theme 1: perception of teachers and students on teenage pregnancy:** knowledge of teachers and students on teenage pregnancy. A good number (26 of 33) of the participants had a good understanding of teenage pregnancy. Regarding the frequency of teenage pregnancy in the community they lived in, most (17 of 25) of the teenagers could not ascertain the incidence of unwanted pregnancy in their community. However, half (4 of 8) of the teachers opined that teenage pregnancy was common in the community among teenagers who had a poor educational background. Conversely, one of the interviewed teachers expressed that the rate of teenage pregnancy is decreasing in society. The following are some of the statements made by the participants: *“It is very common although in our school here maybe around 10% but in a rural area it is also 60% because of lack of enlightenment”* (Teacher from AGS). *“For now it is like it is reducing unlike it was before I think due to awareness”* (Teacher from CHS).

**Causes of teenage pregnancy:** participants' opinion on the causes of teenage pregnancy was sought, and their responses were classified into poverty, lack of home training, poor parental control, child abuse and peer group influences.

**Poverty:** notable from the findings was that more than half (18 of 33) indicated that poverty was a major factor that contributed to teenage pregnancy stating that some girls get pregnant for men hoping to seek financial dependency from them. Some of the observed responses were: *“I can say as a result of poverty, lack of adequate care from the parents, they are unable to take care of their teenage girls, so the girl will now get pregnant for a man”* (Teacher from CHS).

**Peer group influences:** moving into the company of friends who have risky sexual behaviors was also cited as a cause of teenage pregnancy. During the interview one of the students said: *“When someone is having bad friends or peer group it can cause unwanted pregnancy”* (Student from AGS). One of the participants also highlighted waywardness as a cause. *“It is as a result of losing children when they are not taken care of by their parents, or children that are wayward on their own part”* (Teacher from CHS).

**Theme 2: perceived enablers of teenage pregnancy** as regards enablers of teenage pregnancy, a substantial number (18 of 33) of the participants stated that substance abuse, use of illicit drugs, poverty, and peer pressure enabled teenage pregnancy in society. The following are responses from some of the participants: *“When someone is following like bad gang it can cause teenage pregnancy”* (Student from AGS). *“Lack of education, ignorance from the parents”* (Student from CHS). *“I can say mostly poverty, when the girl has no money from home she would look for a man to be taking care of her needs”* (Teacher from CHS). Also, some teachers (4 of 5) mentioned that some family living conditions encourage teenage pregnancy. As some people live in a room apartment, the children observe their parents engaging in sexual activities, hence they desire to engage in sex when they become teenagers. One of the teachers commented thus: ‘´Yes, very well, their family background encourages this thing very well because some parents will have sex when their small children have not slept in the night, they will be romancing themselves.´´

**Knowledge of teachers and students on contraceptives:** the majority (18 of 25) of the students had no knowledge of contraceptives nor did they utilize any form of contraception. On the contrary, all (8 of 8) of the teachers knew what contraceptives were and utilized them at one point or the other.

**Theme 3: solutions to teenage pregnancy**

**Abstinence from sexual activities:** some (9 of 25) of the teenagers suggested that total abstinence from sex remains the most effective way to curb teenage pregnancy. One of the participants during the interview said: *“The teenagers should keep themselves from marital sex, they should walk with good company, they should not partake in bad company, they should not be walking the streets like they don´t have work”* (student from AGS).

**Sex education:** when asked about the solution to teenage pregnancy, about half of the participants emphasized that sex education was important in reducing unwanted pregnancies in society. The following are citations from the participants: *“I like sexual education to be given to them, it is good but we should not say when we are teaching them not to do it but not to go and practice it”* (teacher from CHS). *“We should try to orientate all these children, I use to orientate the boys and girls in my class not to do all these things because of their future, they should keep themselves holy”* (teacher from AHS).

**Government involvement:** less than half (11 of 33) of the participants explained that the government had a major role in reducing the rate of unwanted pregnancy in society. They stated that the government is responsible for creating awareness on of teenage pregnancy and its complications. The participants also suggested that agencies that promote healthy sexual activities among teenagers should be deployed to various schools to educate teenagers on pregnancy. The following are citations from the participants: *“to discourage it from happening and maybe a program or agency controlling the girls just to protect them and agency that will be coming to schools to teach them about pregnancy”* (student from AGS). *“More awareness from the government than even from the school, I think sex education should be introduced, parents should not shy away from their responsibilities”* (teacher from CHS).

**Condom handouts in schools:** almost all (26 of 33) disagreed with condom handouts for teenagers as a means of promoting healthy sexual practices. One of the participants commented thus: *“I don´t believe that they should share it because you are giving the opportunity to go and do that thing”* (teacher from AGS). Conversely, two teachers agreed that sharing condoms would reduce the rate of unwanted pregnancy. *“There is nothing bad in it because even from government awareness on the radio if they go with their contraceptives to prevent unwanted pregnancy. Nothing bad in that”* (teacher from CHS).

## Discussion

In this study, participants reported that a high incidence of teenage pregnancy is noted/observed among girls with low educational levels but has drastically reduced in schools. Our findings suggest that the school was highly protective against teenage pregnancy. This further stressed the importance of girl child education. Similarly, another study [4] stated that low educational level is a risk factor for teenage pregnancy. Moreover, reports have shown that a close relationship exists between teenage pregnancy and low levels of education, stating that a low level of education could be a cause of unwanted pregnancy [15]. Girl child education remains an important tool in combating teenage pregnancy. If we are ever going to succeed in abating teenage pregnancy, it is important that the government provide basic education for every girl child which must be accessible to all rural dwellers. Although one of the teachers in our study observed that teenage pregnancy is common in the urban areas, the teacher further stated that teenage pregnancy is more common in rural areas than in urban areas. Similarly, some studies conducted in Nigeria suggest that teenage pregnancy is more common in rural than urban areas [11,16]; however more studies are needed to confirm this, pertinent to the findings of this study was “poverty” as identified by participants as both a cause and an enabler/enabling factor of teenage pregnancy. The findings of this study are further corroborated by the reports of Ashimolowo *et al*. [5], who opined that teenage pregnancy has often times been found to be a determinant of poverty as the majority of teenage mothers are without a source of livelihood to fend for themselves and the child. Moreover, Kanku and Mash [10] opined that the teenager's early pregnancy was influenced by a desire to have a secure financial support from older men, access to child support grants and poor sexual negotiation skills. Poverty has remained a significant problem in Nigeria, for more than 10 years now, over 100 million people live on less than one dollar a day [17]. Reports showed that as of 2018, Nigeria was the country with the highest number of persons living in extreme poverty [18].

The high rate of unwanted teenage pregnancy in the country has been linked to low socioeconomic conditions of the people [4]. Pregnancy by teenage girls may then be viewed as “wooing” or “a must-do” in order to make ends meet or as a means of survival (meeting economic challenges and to tackle deprivation) as against sexual gratification. If this be the case, as viewed by participants in this study, we can then make a proponent/proposition that, if socioeconomic challenges are tackled in a community like ours, the incidence of teenage pregnancy will significantly drop. Quite despondently, some parents who cannot provide basic needs leave their teenage girls with no option but to fend for themselves, thus promoting sexual activities that unfortunately lead to unwanted pregnancy. Other causes mentioned by participants included poor parenting and peer group influence as major causes of teenage pregnancy. In the same vein, some researchers also highlighted the causes of teenage pregnancy as poor socioeconomic conditions, peer pressure and sexual relationships with older men [2,4,10,11]. Nigeria has been described as a “closeted society” where issues pertaining to sex and contraceptives are not discussed freely [3,19]. As a result, many adolescents have no knowledge or are misinformed about issues relating to sex. Sex education is essential in reducing unwanted pregnancies and teenagers ought to be properly informed about issues relating to sexual activities [20,21]. Although the majority of the study participants in this study opined that there is a need for increased sex education among high school students as a means of preventing unwanted pregnancy, however most of them were not in support of condoms handout in schools because they believe it could increase promiscuity among teenagers. Equally, Akpor and Thupayagale-Tshweneagae [2] also emphasized the need for sex education to curb unwanted pregnancy. Although, some studies have suggested the provision of condoms in secondary schools, as a means of promoting healthy sexual behaviors and reducing the risk of unwanted pregnancy among teenagers [18,22,23]. Similarly, a study conducted in Rwanda also revealed that adolescent sexual practices were considered immoral in Rwanda; thus, the idea of providing condoms in secondary school was rejected [24]. Poor parental control has been linked to an increase risk of teenage pregnancy. Teenage girls have a high tendency to get pregnant if they have limited or no sexual guidance from their parents [4]. Findings from this study revealed that poor parental control is an enabler of teenage pregnancy. Hence, an improved/cordial relationship between parent/guardian and their children is needed to further strengthen the filial cord to facilitate open discourse on sexual issues. In this study, a good number of the participants asserted that government involvement in the fight against teenage pregnancy would go a long way in curbing the scourge. As highlighted in studies conducted by [2,3], government, community and policymakers involvement in programs and initiatives that tackle teenage pregnancy would certainly be a step forward in reducing teenage pregnancy in Nigeria.

**Limitation of the study:** due to the small sample size and the purposive sampling used in selecting study participants in the secondary schools, the results may not be generalizable to a larger context.

**Implication for practice:** this study noted that adequate sex education is still not provided to teenagers; it is paramount that parents and teachers give the right kind of information to teens to prevent misinformation from peers and other sources like friends and social media. Conclusively, the Federal, State, and Local government must participate in fighting the scourge of teenage pregnancy by encouraging the establishment of adolescent-friendly health services.

## Conclusion

Findings from this study revealed that poverty is an active enabler of teenage pregnancy as it encourages teenage girls to consider pregnancy as a means to seek financial stability. Additionally, as reported in this study, teenage pregnancy is predominant among teenagers with low educational background. On this account, it is recommended that girl child education be made affordable and compulsory to break out of the cycle of poverty and teenage pregnancy. There is a need for the government of Nigeria to combat poverty at all levels as a strategy to curb teenage pregnancy.

### What is known about this topic

- Teenage pregnancy remains a major public health issue in Nigeria, with many teenagers being fated to early motherhood resulting in a life filled with turmoil;
- Unwanted pregnancy has been linked to sexual abuse, ignorance, alcohol consumption, substance abuse, cultural traditions, peer pressure and regular sexual intercourse without a reliable contraceptive.

### What this study adds

- Our study revealed that the majority of the participants had knowledge of teenage pregnancy but had limited knowledge of contraceptives, particularly the students;
- Participants identified poverty, substance abuse, lack of parental control, lack of education and peer pressure, as enablers of teenage pregnancy;
- Poverty is an active enabler of teenage pregnancy as it encourages teenage girls to consider pregnancy as a means to seek financial stability.
